# Tailoring the nanoscale morphology of HKUST-1 thin films via codeposition and seeded growth

**DOI:** 10.3762/bjnano.8.230

**Published:** 2017-11-03

**Authors:** Landon J Brower, Lauren K Gentry, Amanda L Napier, Mary E Anderson

**Affiliations:** 1 Hope College, Department of Chemistry, Holland, MI 49422, United States

**Keywords:** atomic force microscopy, copper(II) 1,3,5-benzenetricarboxylate, ellipsometry, surface-anchored metal-organic frameworks

## Abstract

Integration of surface-anchored metal-organic frameworks (surMOFs) within hierarchical architectures is necessary for potential sensing, electronic, optical, or separation applications. It is important to understand the fundamentals of film formation for these surMOFs in order to develop strategies for their incorporation with nanoscale control over lateral and vertical dimensions. This research identified processing parameters to control the film morphology for surMOFs of HKUST-1 fabricated by codeposition and seeded deposition. Time and temperature were investigated to observe film formation, to control film thickness, and to tune morphology. Film thickness was investigated by ellipsometry, while film structure and film roughness were characterized by atomic force microscopy. Films formed via codeposition resulted in nanocrystallites anchored to the gold substrate. A dynamic process at the interface was observed with a low density of large particulates (above 100 nm) initially forming on the substrate; and over time these particulates were slowly replaced by the prevalence of smaller crystallites (ca. 10 nm) covering the substrate at a high density. Elevated temperature was found to expedite the growth process to obtain the full range of surface morphologies with reasonable processing times. Seed crystals formed by the codeposition method were stable and nucleated growth throughout a subsequent layer-by-layer deposition process. These seed crystals templated the final film structure and tailor the features in lateral and vertical directions. Using codeposition and seeded growth, different surface morphologies with controllable nanoscale dimensions can be designed and fabricated for integration of MOF systems directly into device architectures and sensor platforms.

## Introduction

Metal-organic frameworks (MOFs), composed of both metal ions and organic ligands, represent a class of extremely porous, crystalline materials with high surface area. Research has investigated their integration as thin films, namely surface-anchored metal-organic frameworks (surMOFs), into a wide variety of technologies from sensing to low-κ dielectric applications [1–9]. Different morphologies and a range of film thicknesses (10 nm to 100 µm) are required depending on the desired application. For example, rough surfaces present a higher surface area for analytes to access the internal porous networks; and conformal, continuous surfaces are necessary for the incorporation of the MOF within the multilayer stacks commonly implemented for device architectures. Additionally, thin nanoscale films are necessary for the incorporation of surMOFs as dielectric layers and thick microscale films are advantageous for applications in which the MOF pores are utilized for analyte storage.

Layer-by-layer (LBL) solution-phase deposition has been studied for the HKUST-1 system, which consists of Cu(II) ions and trimesic acid (TMA) [10], deposited onto a self-assembled monolayer (SAM) on Au substrates [11–14]. The growth mechanism for HKUST-1 surMOF films fabricated by LBL deposition was found to be Volmer–Weber, with small crystallites nucleating and ripening on the substrate upon continued deposition cycles, as opposed to a van der Merwe growth mechanism that produces a conformal film [11–12]. For surMOF film growth via LBL deposition, it was found that temperature and surface chemistry (terminal functional group of SAM) control the crystal face growth of the crystallites on the substrate [11–12,15–17]. This provides some degree of control over roughness, particle size, surface coverage, and film thickness. In juxtaposition to the LBL method that generated films and crystallites in the sub-100 nm regime, MOF film deposition from mother liquor solutions, which are used to solvothermally produce powders, yield films that have thickness, roughness, and grain sizes on the microscale [2,18–19].

To fabricate the MOF for integration, methods such as microcontact printing and nanografting have been utilized to create chemical patterns onto which the surMOF is selectively grown [20–21]. Confined geometries have been utilized in conjunction with conventional and nonconventional lithography techniques to trap the precursor solution for subsequent solvent evaporation to produce isolated MOF crystallites in predetermined positions [22–24]. Microfluidics and ink-jet printing work in similar manners, delivering the solution according to a predefined design for subsequent MOF crystal formation [25–26]. Processing conditions have been optimized for some specific MOF systems to utilize conventional lithography for patterning of the film [8,27–28]. While these methods offer means to control the spatial location of the MOF for integration, they typically do not present processing parameters to control the morphology of the MOF with regards to nanoscale features such as thickness, roughness, and grain size.

Herein, means for fabricating surMOFs of HKUST-1 via codeposition and seeded growth have been investigated to gain further control over the morphology of these thin films. By varying temperature, time, and deposition method, the goal was to develop and expand design rules to tailor surMOFs with desired thickness, roughness, and grain size. In order to understand the growth mechanism and identify key variables, atomic force microscopy (AFM) and ellipsometry were used to characterize samples, investigating surface morphology, surface roughness, and film thickness.

## Results and Discussion

For this study of codeposition and seeded surMOF film growth, the MOF was anchored to the substrate by a SAM of 16-mercaptohexadecanoic acid (MHDA), which was formed on a thermally deposited gold film on a silicon wafer. To form the HKUST-1 surMOF, this substrate was then immersed in a codeposition solution containing both the inorganic (Cu(II) ions) and organic (trimesic acid) components in dimethyl sulfoxide (DMSO). Deposition time and temperature were studied to understand the surMOF formation. Means for seeding surMOF growth were investigated by combining codeposition and LBL deposition.

### Time study at room temperature (25 °C)

The effect of codeposition time on film thickness was investigated by ellipsometry. Initially, it was hypothesized that this would be a means to control film thickness with potentially thicker films forming after prolonged exposure. The ellipsometric data (Table 1) shows that while the film thickness increased from 0.5 to 1.5 h by almost a factor of two, the film thickness decreased after 5 h and 24 h of deposition and increased after 48 h of deposition. A linear increase in film thickness as a function of time was not observed in contrast to LBL deposition in which film thickness increased as a function of deposition cycles [11]. The film thickness decreases measured for the samples after 5 and 24 h of deposition suggested that the crystallites were not stable after initial formation when the sample was maintained in the DMSO codeposition solution. However, the film thickness increase observed for the 48 h sample may suggest that film growth reoccurred after dissolution of the initial crystallites. This revealed a dynamic process at the interface that affected the amount of MOF anchored to the substrate. Ellipsometry, with its laser beam spot size of ca. 1 mm, allowed for fast and efficient sampling across the entire substrate. In contrast to AFM with sampled region sizes on the micro- and nanoscale, ellipsometry provided a more global overview of the film than the local sampling of the AFM. AFM has been integral to mapping out the nanoscale morphology of surMOF thin films as well as identifying features formed on the surface of MOF crystals [11–12,29–32].

**Table 1:** Average film thickness and roughness values along with standard deviations observed for specified codeposition conditions.

temperature (°C)	time (h)	thickness (nm)	roughness (nm)

25	0.5	4.08 ± 0.45	11.8 ± 2.2
	1.5	7.6 ± 1.3	19.3 ± 5.6
	5	4.8 ± 1.5	14.9 ± 2.0
	24	3.08 ± 0.79	15.6 ± 2.6
	48	6.22 ± 0.88	4.5 ± 2.3
35	1.5	5.9 ± 1.4	10.7 ± 3.0
	5	12.3 ± 1.2	21.3 ± 6.5
50	1.5	5.9 ± 1.4	11.8 ± 4.7
	5	5.5 ± 1.0	6.3 ± 1.3
75	1.5	11.4 ± 2.4	10.0 ± 4.0
	5	4.6 ± 2.4	5.7 ± 2.0

**Figure 1:**
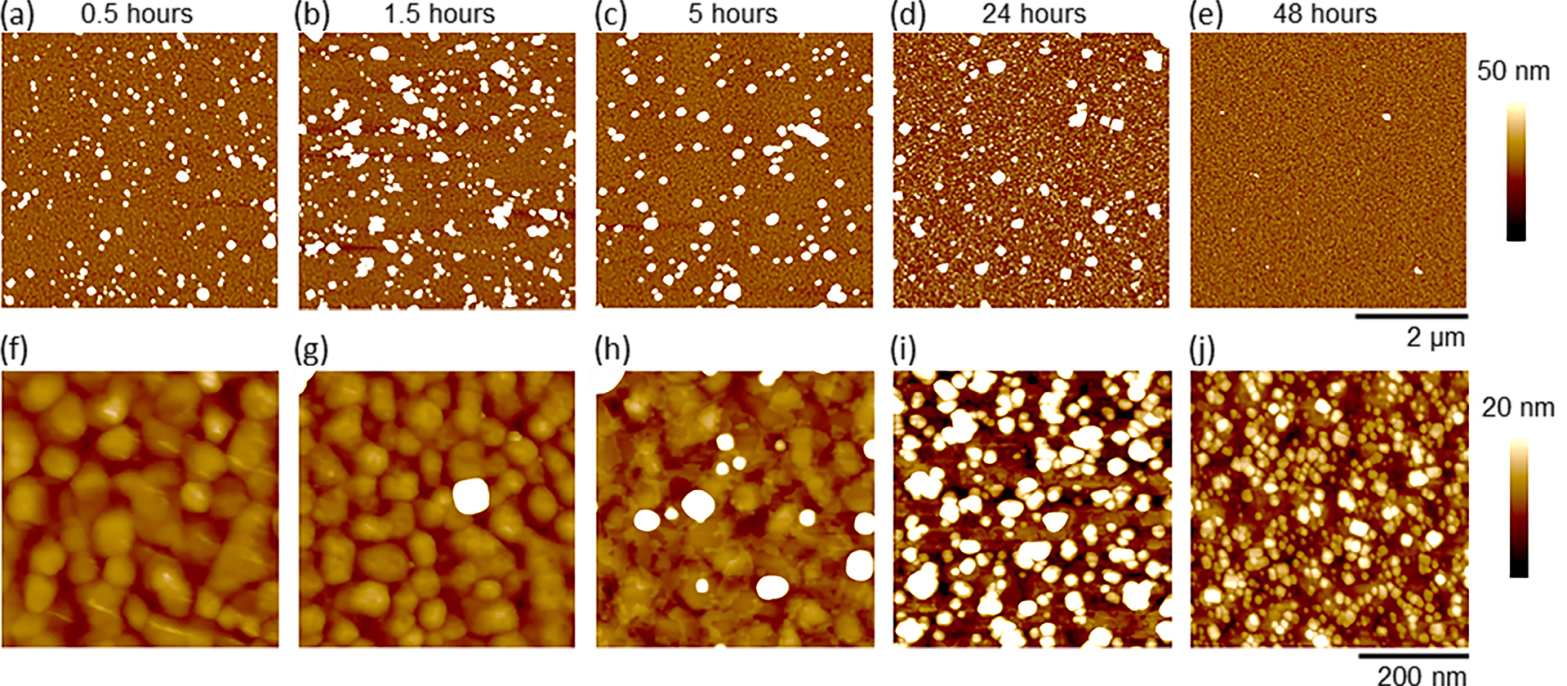
Representative AFM images of HKUST-1 surMOFs fabricated via codeposition at 25 °C on SAM-coated Au surfaces. Samples were synthesized over varied lengths of time (as indicated above each column of images). Shown in (a–e) are 5 μm × 5 μm images set to the same *z*-scale (50 nm) and shown in (f–j) are 500 nm × 500 nm images set to the same lower *z*-scale (20 nm) to visually render the smallest particles on the substrate. The higher magnification images were taken in regions between the largest MOF crystallites and selected specifically to characterize the smallest crystallites nucleated on the surface. Note the gold grain structure in the background of these higher resolution images.

AFM was employed to investigate how the morphology of the film changed as a function of the deposition time. Representative images for the different time points at room temperature are shown in Figure 1 with the average film roughness (*R*q) given in Table 1. Between the time points of 0.5 and 1.5 h (Figure 1a,b), the feature size of the crystallites and the average film roughness increased (from 11.8 ± 2.2 nm to 19.3 ± 5.6 nm) corresponding with increased surface coverage that reflected the ellipsometrically observed film thickness increase. When the deposition time was increased to 5 and 24 h (Figure 1c,d), fewer large particles were observed and the average observed film roughness decreased slightly (from 19.3 ± 5.6 nm after 1.5 h to 14.9 ± 2.0 nm and 15.6 ± 2.6 nm after 5 and 24 h, respectively). This corresponded to the decreased average film thickness observed by ellipsometry (from 7.6 ± 1.3 nm after 1.5 h to 4.8 ± 1.5 nm and 3.08 ± 0.79 nm after 5 and 24 h, respectively). In addition at these time points (5 and 24 h), the presence of smaller particles between the larger particles became prevalent, as is shown in the higher magnification images (Figure 1h,i). After 48 h of deposition (Figure 1e,j), the film had a very high coverage of small (predominantly sub-10 nm height) crystallites (most clearly seen in Figure 1j) consistent with the decrease in film roughness to a third (from 15.6 ± 2.6 nm after 24 h to 4.5 ± 2.3 nm after 48 h). The film thickness after 48 h (6.22 ± 0.88 nm) increased above the 24 h sample (3.08 ± 0.79 nm) and was within error the same as the 1.5 h (7.6 ± 1.3 nm) and 5 h (4.8 ± 1.5 nm) samples. The similarity of the ellipsometric film thicknesses with significantly different feature sizes (quantitatively shown as a three- and four-fold difference in film roughness) suggested that while deposition time could not control film thickness, it could tune film morphology.

### Time and temperature study

In addition to codeposition at 25 °C, three addition temperatures were investigated (35 °C, 50 °C, 75 °C) at time points of 1.5 and 5 h. These two time points were selected for this investigation because they were distinctly different from one another in the 25 °C samples. The 1.5 h sample at 25 °C had the highest thickness and roughness values. The 25 °C sample submerged for 5 h had a marked decrease in thickness and was the initial time point at which the proliferation of small particles was observed. In addition, durations of 1.5 and 5 h were reasonable time lengths for chemical processing. It was postulated that an increase in temperature could increase film thickness or accelerate the dynamic process observed at room temperature.

Deposition at higher temperatures did indeed produce thicker films than were observed for the different time conditions investigated at room temperature. For the 1.5 h time point, ellipsometry showed that the thickest film (11.4 ± 2.4 nm) occurred at the highest temperature (75 °C). At the lower temperatures for the 1.5 h duration, lower film thicknesses consistent within error were found (7.6 ± 1.3 nm, 5.9 ± 1.4 nm, and 5.9 ± 1.4 nm). In contrast for the 5 h time point, the thickest film (12.3 ± 1.2 nm) was found for the film fabricated at 35 °C. The other films were found to have thicknesses again consistent within error (4.8 ± 1.5 nm, 5.5 ± 1.0 nm, and 4.6 ± 2.4 nm).

**Figure 2:**
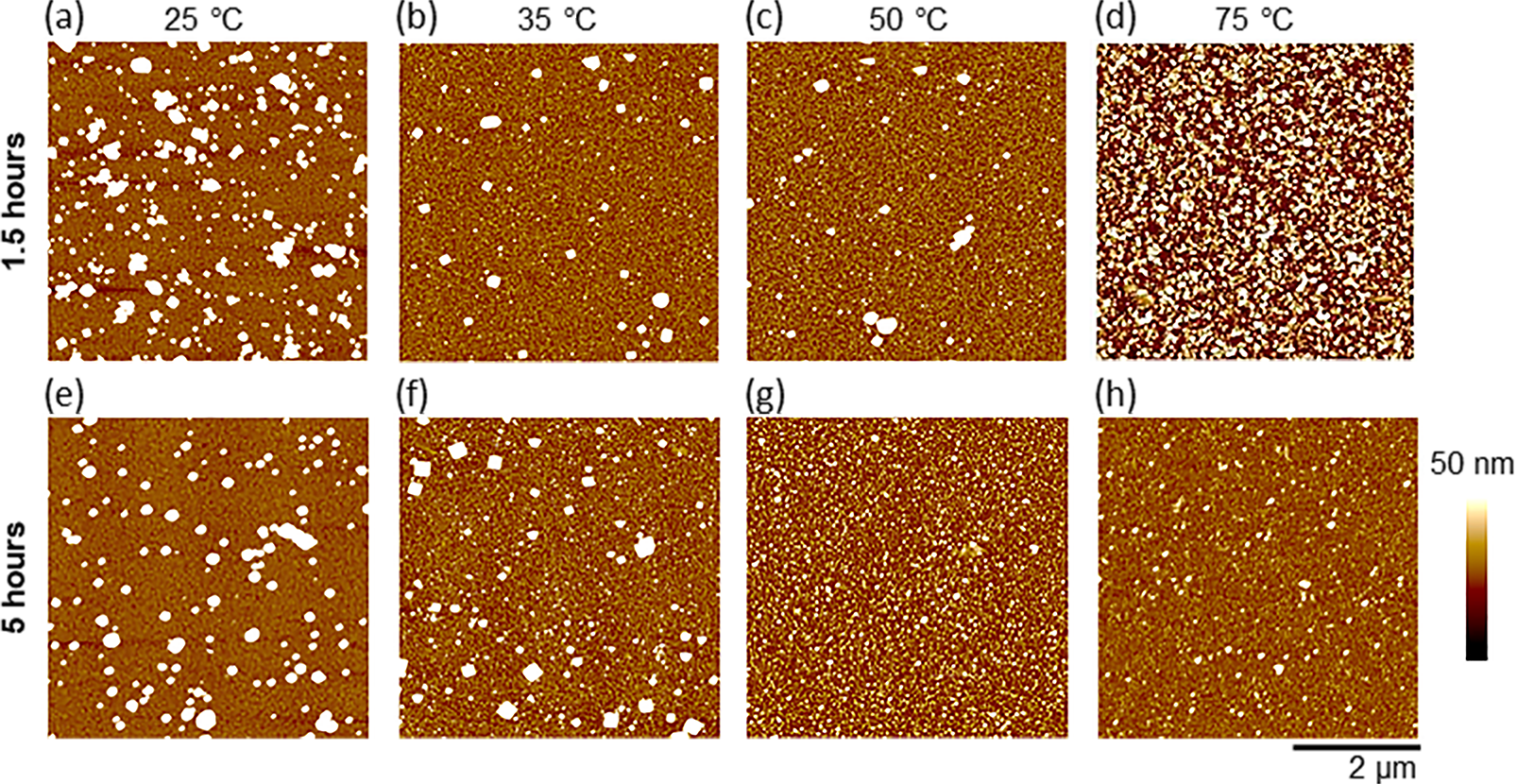
Representative AFM images (5 μm × 5 μm) of HKUST-1 surMOFs fabricated via codeposition at different temperatures (as indicated above each column of images) on SAM-coated Au surfaces. Samples were exposed for different durations; either 1.5 h (a–d) or 5 h (e–h). All images were set to the same *z*-scale (50 nm) for visual comparison.

To explore how the morphology of these films was affected by deposition at higher temperatures, AFM images were collected (Figure 2). The 35 °C and 50 °C samples after 1.5 h were indistinguishable regarding film thickness and roughness. The AFM images (Figure 2b,c) show similar morphologies composed of large particles with small particles being observable. Note that these small particles were absent at 25 °C (Figure 2a). After 5 h at 35 and 50 °C, these samples that were quite similar became distinctly different. This is especially apparent in the AFM images (Figure 2f,g), as well as in the average film thickness that doubled for the 35 °C sample (from 5.9 ± 1.4 nm to 12.3 ± 1.2 nm) and remained unchanged for the 50 °C sample (at 5.9 ± 1.4 nm and 5.5 ± 1.0 nm). These 35 °C and 50 °C samples after 5 h of deposition had distinct morphologies and roughnesses that mirrored samples deposited at 25 °C for 24 and 48 h, respectively. This suggested that the same dynamic process was occurring. However, it was accelerated by the elevated temperatures. Further support for this was seen in comparing the 50 °C and 75 °C sample after 1.5 h of deposition. A higher coverage of particles is apparent in the AFM image of the 75 °C sample (Figure 2d), which reflected the observation that the film thickness for the 75 °C sample was double that of the 50 °C sample. This also paralleled the doubling of film thickness observed at room temperature for the 48 h sample relative to the 24 h sample.

For the implementation of surMOFs into most potential applications, it is necessary for the films to be continuous across the substrate and have controllable thicknesses. Preliminary investigations found that at lower concentrations of the codeposition solution, less material was anchored to the substrate. It would follow that at higher concentration, one could increase the film thickness. However, the solubility of the reagents within the solution was prohibitive to investigating higher concentration. Additionally, preliminary work found that significant film formation neither occurred when copper acetate was used as the metal ion source, nor when ethanol was used as the solvent. Furthermore, initial experiments showed that the codeposition solution with dimethylformamide as the solvent resulted in a similar dynamic surface process. However, the initial large particles that occurred were smaller relative to those observed using DMSO at the early time points. Future experiments may investigate the effect of altering the ratio of the metal ion and organic component.

### Seeded growth

Film morphology could be tailored by codeposition utilizing time and temperature as variables to tune the structure. While an increased film thickness was found at elevated temperatures, the upper bound of film thicknesses for codeposition seems limited (Table 1) and did not result in a continuous film across the substrate. It was hypothesized that the film thickness could be increased by LBL deposition on top of samples with foundational surMOF crystallites formed by codeposition.

To investigate whether the underlying morphology of the codeposited seed layer crystallites could be maintained throughout the LBL deposition process, codeposited samples with unique surface morphologies were identified. The room-temperature study (25 °C) investigated samples exposed to the codeposition solution for different durations and revealed that distinct morphologies could be controlled by tuning exposure times. The 1.5 h and 48 h samples had similar thicknesses (7.6 ± 1.3 nm and 6.22 ± 0.88 nm), but very different morphologies. Qualitatively, the morphology of the 1.5 h sample had lower surface coverage with larger particles relative to the 48 h sample. Quantitative analysis of AFM images showed that the roughness of the 1.5 h sample was four times that of the 48 h sample. While this type of control of surface morphology has potential, the time requirements for the smooth film could be prohibitive. The temperature study confirmed that the same dynamic process resulting in distinct morphologies at room temperature could be accelerated by elevating the temperature. That is, the morphology, roughness, and thickness found after 48 h for the 25 °C sample could be achieved more readily after 5 h at 50 °C. (For comparison, a representative AFM 500 nm × 500 nm image of the sample codeposited for 5 h at 50 °C can be found in Supporting Information File 1.)

**Figure 3:**
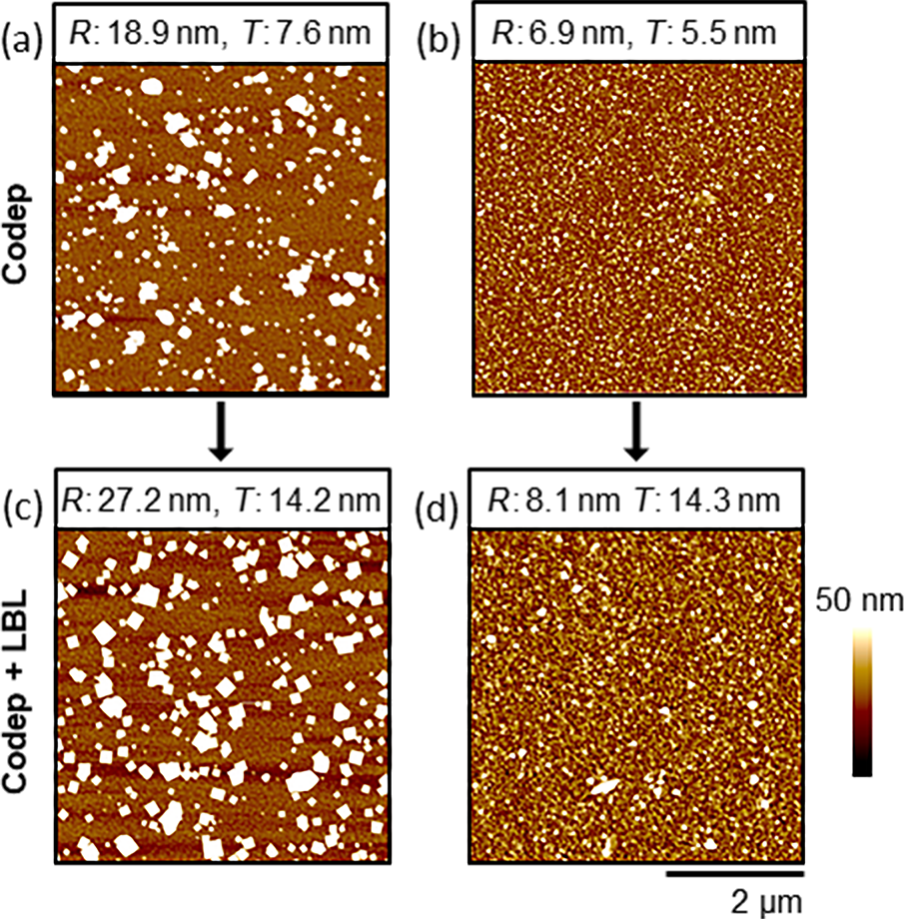
Representative AFM images (5 μm × 5 μm) of HKUST-1 surMOFs fabricated via codeposition (codep) at 25 °C for 1.5 h (a) and 50 °C for 5 h (b) on SAM-coated Au surfaces. Additional layers of HKUST-1 were added to these codeposited samples via layer-by-layer (LBL) deposition. The subsequent surface morphology was imaged (c,d) and the previous surface morphology was maintained. Data regarding the roughness (*R*) for the image shown here and average film thickness (*T*), as measured by ellipsometry, are provided above the images for comparison. All images were set to the same *z*-scale (50 nm).

To potentially template film morphologies, samples seeded with unique surface morphologies were fabricated by codeposition for 1.5 h at 25 °C and 5 h at 50 °C (Figure 3a,b). These two conditions produced films with similar thicknesses, yet with different morphologies that were shown quantitatively to have had roughnesses of 19.3 ± 5.6 nm and 6.3 ± 1.3 nm at 25 °C and 50 °C, respectively. These films were then taken in parallel through four LBL deposition cycles. The film thickness in both of these cases increased by ca. 7 nm, which was consistent with four cycles of deposition on a MHDA SAM-coated substrate [11]. After this LBL process, surface morphologies were consistent with that of the underlying seed crystallites (Figure 3c,d). These two films fabricated by LBL deposition on top of films seeded by codeposition had similar average film thicknesses (14 nm). However, they had significantly different average film roughnesses with 32.8 ± 14.2 nm and 12.8 ± 5.7 nm observed for the films deposited on the substrate seeded by using codeposition for 1.5 h at 25 °C and for 5 h at 50 °C, respectively.

This research shows that LBL deposition on substrates seeded with crystallites formed by codeposition could result in thicker films and maintain tailored morphologies. This control over film thickness and morphology is important for the integration of MOFs into a range of thin film architectures. In contrast to the successful seeding via codeposited crystals for subsequent LBL deposition, initial attempts to use surMOF films formed by LBL as seed crystallites for codeposition were unsuccessful. In this case, neither increased film thicknesses nor preservation of the initial film morphology was observed.

Associated with the studies herein, dropcasting on substrates seeded by codeposition or LBL deposition was investigated. Dropcasting a solution (containing the inorganic and organic components of the MOF) onto a substrate followed by heating to eliminate the solvent and crystallize the film is a common method for the formation of continuous, albeit thick, MOF films. To form continuous films across a substrate, high solution concentrations are required and these result in thicknesses commonly on the micrometer-scale. Preliminary investigations using seeded surMOF films formed by codeposition or LBL were effective for fabricating conformal, continuous, and thinner films from more dilute dropcast solutions. Future research will further optimize this process by controlling solution concentration, temperature, and atmospheric conditions to permit the formation of sub-micrometer, conformal films.

## Conclusion

Films formed by codeposition were similar to those formed by LBL in that they were composed of nanocrystallites and were not conformal films produced by a van der Merwe growth mechanism. However, the Volmer–Weber growth mechanism (with crystallite nucleation and ripening) that was observed for the LBL deposition was not observed in the same manner for the codeposition. Throughout the codeposition procedure, a dynamic process was observed at the substrate interface. Large particles initially formed on the substrate, followed by the increased prevalence of smaller crystallites alongside the disappearance of the larger particles, and finally the substrate became covered with a high density of small (ca. 10 nm) crystallites. Altering deposition time and temperature was found to control size and density of the particles on the surface, resulting in films with distinctly different morphologies and surface roughnesses. Elevated temperatures were found to expedite the film formation, thus obtaining the full range of surface morphologies within reasonable time frames. Initial morphological properties of the codeposited films were conserved when performing the LBL deposition process on substrates that were seeded under two different codeposition conditions.

## Experimental

### Materials

Trimesic acid (TMA, 95%), dimethyl sulfoxide (DMSO, Aldrich, spectrophotometric grade), and 16-mercaptohexadecanoic acid (MHDA, 90%) were obtained from Aldrich (St. Louis, MO, USA). The DMSO was purged with nitrogen and passed through columns of molecular sieves. Copper(II) nitrate hemi(pentahydrate) (ACS grade) and copper(II) acetate monohydrate were received from Fisher Scientific (Fair Lawn, NJ, USA). Absolute, anhydrous ethyl alcohol (200 proof, ACS/USP grade) was attained from Pharmco-Aaper (Shelbyville, KY, USA). All chemicals were used as received, unless otherwise noted. Gold substrates were obtained from Platypus Technologies (New Orleans, LA) in the form of silicon wafers with a 5 nm titanium adhesion layer and 100 nm of gold.

### Methods

**Substrate Preparation:** HKUST-1 surMOF films were fabricated by the codeposition of TMA and copper ions onto a gold substrate previously functionalized by a self-assembled monolayer (SAM) that consisted of MHDA. The gold substrate was first fully immersed in approximately 10 mL of a 1 mM MHDA ethanol solution for 1 h, which formed the foundational anchor for the framework. Once removed from solution, the sample was rinsed thoroughly with ethanol and dried with nitrogen gas.

**Codeposition SurMOF formation:** The codeposition solution was prepared, consisting of 0.53 M copper nitrate and 0.27 M TMA in DMSO. This concentration was half of the typical solution from which MOF powders were crystallized [10,22]. The codeposition solution was sonicated and stirred for 5 min, after which approximately 10 mL were used to submerge the substrate. Following submersion, a hotplate was used to achieve and maintain temperatures above 25 °C for the duration of the deposition process. The sample was then removed from solution, rinsed with ethanol, dried with nitrogen, and stored in a dry box.

**Layer-by-Layer SurMOF formation:** The LBL deposition of surMOF on a gold substrate functionalized by a SAM was fabricated according to the literature by alternating, solution-phase deposition [11]. This process was automated by a Midas III automated slide stainer. For all experiments herein, solutions were held at room temperature.

### Characterization

All samples were characterized by atomic force microscopy (Figures 1–3) and ellipsometry (Table 1). In addition, characterization by infrared spectroscopy was conducted to confirm composition, and representative data is presented in Supporting Information File 1 (Figure S2) [13–14].

**Atomic force microscopy:** Multiple images (512 × 512 pixels) were obtained for each sample at 5 μm × 5 μm and 500 nm × 500 nm and used a Dimension Icon atomic force microscope (Bruker, Santa Barbara, CA, USA), which was operated in peak force tapping mode. Etched silicon tips, SCANASYST-AIR (Bruker, Santa Barbara, CA, USA), with a spring constant range of 0.2–0.8 N/m and a resonant frequency range of 45–95 kHz were used. Scan parameters were as follows: 1 Hz scan rate, 12 μm *z*-range, 250 mV amplitude set point, and 100 mV drive amplitude. AFM data presented herein are representative of the compilations of data specific to each sample set.

**Image analysis:** Image analysis was routinely carried out using the Nanoscope Analysis software (Bruker, Santa Barbara, CA, USA). This program was used to appropriately flatten and scale the image. The geometric average surface roughness, *R*q, was calculated for each image. The reported roughness values and standard deviations herein (Table 1) reflect the average *R*q from a minimum of three images taken per sample at 5 μm × 5 μm.

**Ellipsometry:** To investigate film growth, film thickness was characterized by using a variable-angle discrete wavelength ellipsometer (PHE-101 VADE, Angstrom Advanced, Braintree, MA). Note that the film thickness determined by ellipsometry is an average of the thickness of particulates within the samples region of the 1 mm laser spot size. The use of proximal probes in addition to optical methods to characterize these types of films has been highlighted previously in the literature [11]. Data were acquired for each sample and collected from a minimum of five areas at a wavelength of 632.8 nm and fixed angle of 70°. The PHE-101 analysis software used the following refractive index values to calculate film thickness for the gold substrate: *n*_s_ = 0.148 and *k*_s_ = 3.594 and for the organic thin film: *n*_f_ = 1.5 and *k*_f_ = 0. The average film thickness and standard deviations are reported in Table 1.

## Supporting Information

Supporting Information features a representative image data set (500 nm × 500 nm) for the samples codeposited for 5 h at 50 °C and for 5 h at 75 °C, as well as representative IR spectra for samples produced by codeposition and seeded growth.

File 1 Additional experimental data.
