# Supplementary material for: Tailoring the nanoscale morphology of HKUST-1 thin films via codeposition and seeded growth
Source: Beilstein J Nanotechnol. 2017 Nov 3;8:2307–14. doi: 10.3762/bjnano.8.230 (PMC5687001; doi:10.3762/bjnano.8.230)
Supplement: File 1 — Additional experimental data. [file Beilstein_J_Nanotechnol-08-2307-s001.pdf]

Supporting Information

for

Tailoring the nanoscale morphology of HKUST-1 thin films via codeposition and seeded growth

Landon J. Brower, Lauren K. Gentry, Amanda L. Napier and Mary E. Anderson*

Address: Hope College, Department of Chemistry, Holland, MI 49422, United States

Email: Mary E. Anderson* - meanderson@hope.edu

* Corresponding author

Additional experimental data

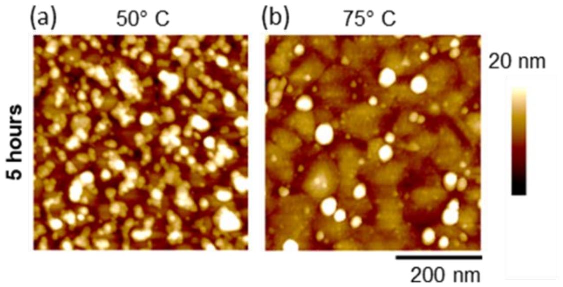

Figure S1: AFM images (500 nm × 500 nm) of HKUST-1 surMOFs fabricated for 5 h via codeposition at (a) 50 °C and (b) 75 °C on SAM-coated Au surfaces. These images at a higher magnification than those shown in Figure 2 allow for the clear visualization of the nanoscale MOF crystallites. Both images were set to the same *z*-scale (20 nm).

Infrared spectroscopy: IR spectra collected from 3800–600 cm^{-1} in the attenuated total reflectance (ATR) mode of a Thermo Scientific Nicolet iS50 instrument. The spectral resolution was 4 cm^{-1} and a bare gold substrate was used as the background for the surMOF films.

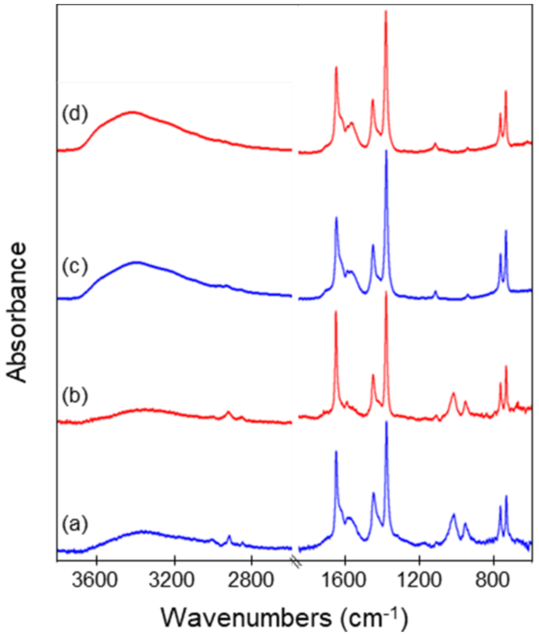

Figure S2: IR spectra of HKUST-1 surMOFs fabricated via codeposition (a) at 25 °C for 1.5 h and (b) at 50 °C for 5 h on SAM-coated Au surfaces. Four additional layers of HKUST-1 were added to these codeposited samples via layer-by-layer deposition. The subsequent IR spectra are shown in (c,d).
